# TET1 Deficiency Impairs Morphogen-free Differentiation of Human Embryonic Stem Cells to Neuroectoderm

**DOI:** 10.1038/s41598-020-67143-x

**Published:** 2020-06-25

**Authors:** Hanqin Li, Zhixing Hu, Houbo Jiang, Jiali Pu, Ilana Selli, Jingxin Qiu, Baorong Zhang, Jian Feng

**Affiliations:** 1 Department of Physiology and Biophysics, State University of New York at Buffalo, Buffalo, NY 14203 USA; 2 Department of Neurology, Second Affiliated Hospital, College of Medicine, Zhejiang University, Hangzhou, China; 3 Department of Pathology and Laboratory Medicine, Roswell Park Comprehensive Cancer Center, Buffalo, NY 14263 USA

**Keywords:** Neural stem cells, Stem-cell differentiation

## Abstract

The TET family of 5-methylcytosine (5mC) dioxygenases plays critical roles in development by modifying DNA methylation. Using CRISPR, we inactivated the TET1 gene in H9 human embryonic stem cells (hESCs). Mutant H9 hESCs remained pluripotent, even though the level of hydroxymethylcytosine (5hmC) decreased to 30% of that in wild-type cells. Neural differentiation induced by dual SMAD inhibitors was not significantly affected by loss of TET1 activity. However, in a morphogen-free condition, TET1 deficiency significantly reduced the generation of NESTIN^+^SOX1^+^ neuroectoderm cells from 70% in wild-type cells to 20% in mutant cells. This was accompanied by a 20-fold reduction in the expression level of PAX6 and a significant decrease in the amount of 5hmC on the PAX6 promoter. Overexpression of the TET1 catalytic domain in TET1-deficient hESCs significantly increased 5hmC levels and elevated PAX6 expression during differentiation. Consistent with these *in vitro* data, PAX6 expression was significantly decreased in teratomas formed by TET1-deficient hESCs. However, TET1 deficiency did not prevent the formation of neural tube-like structures in teratomas. Our results suggest that TET1 deficiency impairs the intrinsic ability of hESCs to differentiate to neuroectoderm, presumably by decreasing the expression of PAX6, a key regulator in the development of human neuroectoderm.

## Introduction

The utility of human pluripotent stem cells (hPSCs) including human Embryonic Stem Cells (hESCs) lies in their abilities for unlimited self-renewal and their potential to differentiate to all types of somatic cells^1^ for many applications including cell replacement therapies^2^. Directed differentiation of hPSCs to a particular cell type of interest is an epigenetic conversion process^3–5^. Thus, understanding epigenetic mechanisms in the differentiation of hPSCs is crucial for deciphering human development and improving the generation of inaccessible types of human cells *in vitro*. DNA methylation is one of the major mechanisms in epigenetics regulation^6^, by contributing to the specification of local chromatin state^7^, regulating gene expression^8,9^ and cooperating with histone modifications^10–12^. 5-methylcytosine (5mC) represents the main and the best characterized component of DNA methylation in mammals^6,13,14^. Active demethylation of 5mC requires a series of oxidation reactions catalyzed by the ten-eleven translocation (TET) family of dioxygenases^15–17^, which sequentially oxidize 5-methylcytosine (5mC) to 5-hydroxymethylcytosine (5hmC)^15^, then to 5-formylcytosine (5fC) and 5-carboxylcytosine (5caC)^18^. The latter two derivatives can be recognized and repaired to cytosine by the DNA repair system, thus functionally restoring 5mC to C through this process^19^. The critical roles of TET proteins in regulating DNA methylation and hence the epigenetic state of a cell have stimulated intensive studies of their functions in many important cellular processes in development and disease^20,21^.

Among the three members of the TET family, TET1 is the most abundant in ESCs^22,23^. Several studies have generated Tet1 loss-of-function mouse ESCs^22,24^ or Tet1 knockout mice^25,26^. However, phenotypes reported in these studies have marked variations, ranging from failure in maintaining pluripotency *in vitro*^24^, to skewed lineage commitment in differentiation^22^, early embryonic lethality^26^, or generally normal development^25^ with impaired hippocampal neurogenesis^27^. The highly variable phenotypes may be related to mouse genetic background and breeding history^26^. The conflicting phenotypes in mouse and the important role of human TET1 in epigenetic reprogramming of human fibroblasts to neurons^28^ motivated us to study the consequence of TET1 loss in human. In this study, we generated TET1-deficient H9 hESCs by inactivating the catalytic activity of TET1 using the CRISPR/Cas9 system^29^. TET1-deficient hESCs maintained pluripotency but exhibited impaired differentiation to neuroectoderm and neurons in a morphogen-free condition. The 5hmC level on PAX6 promoter was significantly decreased, as was the expression of PAX6, a critical regulator of neuroectoderm development in human^30^. Overexpression of TET1 catalytic domain in TET1-deficient hESCs rescued the defects in 5hmC levels in hESCs and PAX6 expression during differentiation. Consistent with these, teratomas derived from TET1-deficient hESCs showed a significant decrease in PAX6 expression, but contained neural tube-like structures. The study reveals a critical function of TET1 in the differentiation of hESCs to neuroectoderm and neurons.

## Results

### CRISPR-mediated Inactivation of TET1 in H9 hESCs

**Figure 1:**
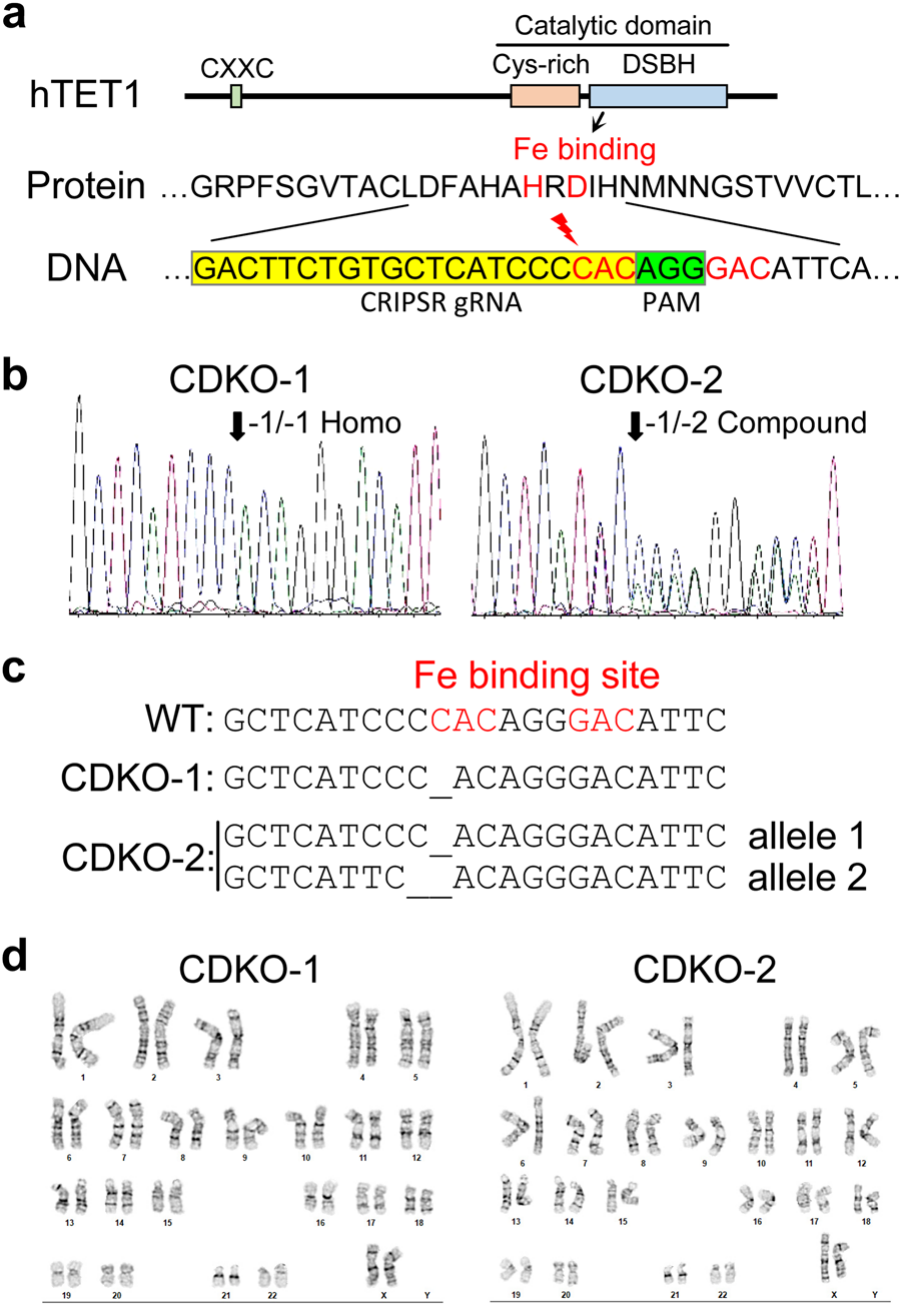
CRISPR-mediated mutations of catalytic domain of TET1 in H9 hESCs. (**a**) Double strand break was introduced by CRISPR/Cas9 before the first iron binding site to induce non-homologous end joining in TET1 gene. (**b**) Sequencing traces of PCR products amplified from genomic DNA of two hESC clones (CDKO-1 and CDKO-2), showing frame shift mutations in both alleles in each clone. (**c**) Sequencing of cloned PCR products showing homozygous 1 bp deletion in CDKO-1 clone and compound heterozygous deletions of 1 bp and 2 bp in CDKO-2 clone. The frameshift mutations destroy iron-binding sites and downstream catalytic domain. (**d**) Normal karyotype for the two TET1 mutant clones.

As the catalytic activity of TET1 requires iron binding^15^, we designed a guide RNA to introduce a double-stranded break before the first iron-binding site in the catalytic domain of human TET1 (Fig. 1a). After CRISPR/Cas9-mediated gene targeting in H9 hESCs, two independent clones with a frame shift mutation in each allele of TET1 were obtained. One clone (CDKO-1) carried homozygous deletion of 1 base pair (bp) in the first iron-binding site of each allele of TET1 (Fig. 1b,c), generating a frame shift mutation that created a stop codon 24 bp downstream. The other clone (CDKO-2) had 1 bp deletion on one allele and 2 bp deletion on the other allele of TET1 (Fig. 1b,c), generating stop codons 24 bp and 20 bp downstream, respectively. Both lines of mutant H9 hESCs had normal karyotypes (Fig. 1d).

**Figure 2:**
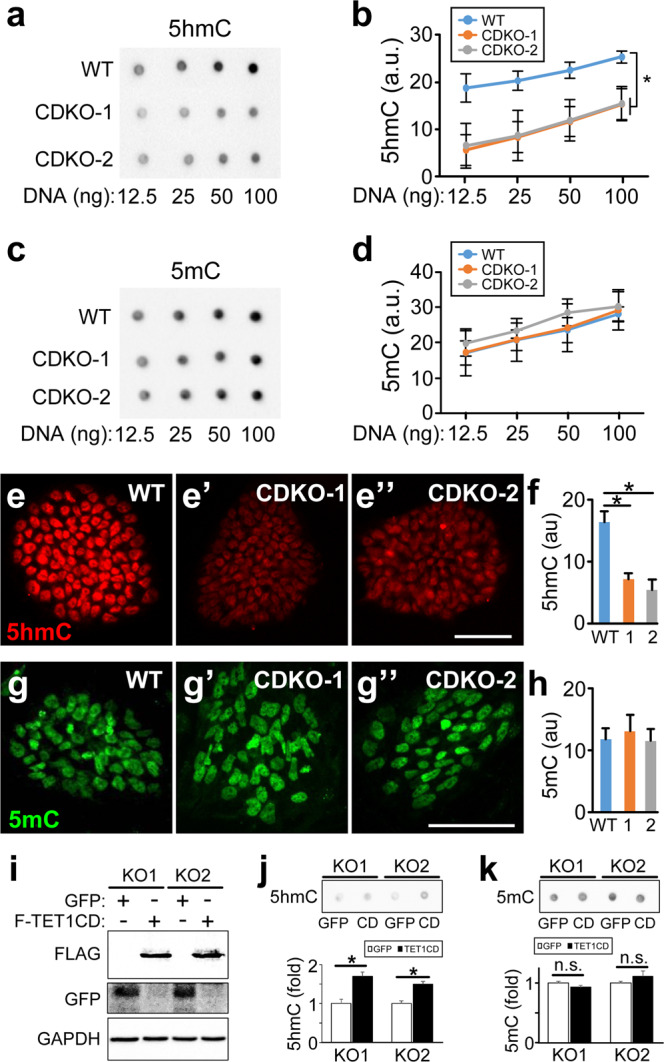
Reduced 5hmC level in TET1-deficient hESCs. (**a**,**b**) Dot blot (**a**) and quantification (**b**) of 5hmC in WT and TET1-deficient hESCs. *p < 0.001, paired Student’s *t*-test, n = 3. (**c**,**d**) Dot blot (**c**) and quantification of 5mC in WT and TET1-deficient hESCs. Full images of the dot blots without any imaging processing are displayed in (**a**) and (**c**). (**e**,**f**) 5hmc staining (**e**-**e”**) and quantification (**f**) in WT (**e**) and TET1-deficient (e’ and e”) hESCs. *p < 0.001, Student’s *t*-test, n = 5. (**g**,**h**) 5mC staining (**g-g”**) and quantification (**h**) in WT (**g**) and TET1-deficient (**g’** **g”**) hESCs. Scale bar, 100 µm. (**i**,-**k**) The two TET1 mutant hESC lines (KO1 and KO2) were infected with lentivirus expressing GFP or FLAG-tagged TET1 catalytic domain (F-TET1CD). Total cell lysates were blotted with the indicated antibodies (**i**). Genomic DNA isolated from these cells were dot-blotted with antibodies against 5hmC (**j**) or 5mC (**k**). *p < 0.05, paired Student’s *t*-test, n = 4.

To examine the effect of TET1 mutations on DNA methylation and hydroxymethylation, we performed dot blot analysis of genomic DNA isolated from wild-type (WT) and the two mutant lines of H9 with antibodies against 5-hydroxymethylcytocine (5hmC) and 5-methylcytosine (5mC). There was a significant reduction in 5hmC level in the two mutant lines, to approximately 30% of the level in wild-type H9 (Fig. 2a,b) (30% ± 7% for CDKO-1, 34% ± 10% for CDKO-2, calculated by the intensity of the dots containing 12.5 ng DNA). This reduction is more severe than what has been observed in Tet1 deficient mouse ESCs, where 60% of 5hmC remained^22,25^. TET1 mutations in H9 hESCs generated no significant change in 5mC level (Fig. 2c,d), in agreement with previous studies on Tet1 deficient mESCs^25^. In human pluripotent stem cells, 5mC accounts for 8% of total cytosine, while 5hmC only accounts for 0.12% of total cytosine, as measured by mass spectrometry^31^. Thus, even a significant reduction in 5hmC level may not lead to a significant change in the amount of its precursor, 5mC. Immunostaining confirmed the significant reduction of 5hmC levels (Fig. 2e,f) and unchanged 5mC levels (Fig. 2g,h) in the two TET1 mutant lines, in comparison to wild-type H9 hESCs.

The ability of TET1 to catalyze the oxidation of 5mC to 5hmC is dependent on the catalytic domain (CD)^15^. TET1 is a large gene with 13 exons encoding a protein of 2136 amino acids. Thus, we inactivate TET1 by mutating the catalytic domain. To test whether the catalytic activity of TET1 is important for the phenotypes that we observed, we overexpressed FLAG-tagged TET1 catalytic domain (FLAG-TET1CD) in the two TET1 mutant hESC lines (CDKO1 and CDKO2) using lentivirus. Overexpression of FLAG-TET1CD (Fig. 2i) significantly increased 5hmC levels in the two mutant hESC lines (Fig. 2j) without changing 5mC levels significantly (Fig. 2k).

#### TET1 Deficiency does not affect spontaneous differentiation of hESCs

**Figure 3:**
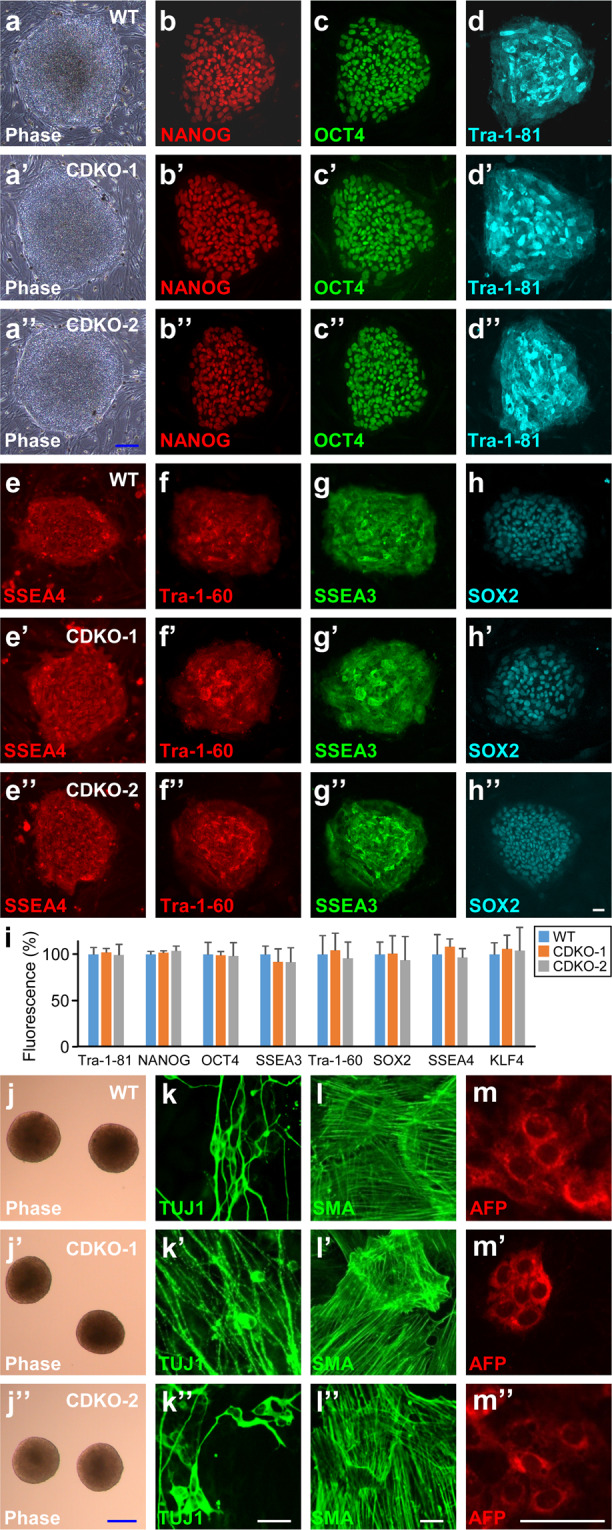
Pluripotency maintained in TET1-deficient hESCs. (**a–i**) Phase contrast images (**a**-**a**”) and immunostaining of indicated pluripotency markers (**b**-**h”**) in wild-type (**a–h**) or TET1-deficient (**a’-h”**) H9 hESCs. Fluorescence intensity was quantified from at least five independent colonies for each condition and normalized against the values in WT (**i**). (**j–m”**) Wild-type (**j–m**) or TET1-deficient (**j’**-**m”**) H9 hESCs were differentiated spontaneously in serum-containing medium through embryoid bodies (**j–j”**) to cells of all three germ layers, as indicated by immunostaining for the ectoderm marker TUJ1 (**k**-**k”**), the mesoderm marker SMA (**l**-**l”**) and the endoderm marker AFP (**m**-**m”**). Blue bars, 100 µm; white bars, 25 µm.

TET1-deficient hESCs exhibited normal primed state morphology as the wild-type H9 cells (Fig. 3a-a”) and expressed pluripotency markers, such as NANOG (Fig. 3b-b”), OCT4 (Fig. 3c-c”), Tra-1-81 (Fig. 3d-d”), SSEA4 (Fig. 3e-e”), Tra-1-60 (Fig. 3f-f”), SSEA3 (Fig. 3g-g”) and SOX2 (Fig. 3h-h”), to similar degrees as those in H9, as shown in the quantification of fluorescence intensities (Fig. 3i). TET1-deficient hESCs formed embryoid bodies (EBs) with normal morphology in suspension culture without bFGF (Fig. 3j-j”). In spontaneous differentiation in serum-containing medium, TET1-deficient EBs generated cells of all three germ layers, such as TUJ1^+^ ectoderm cells (Fig. 3k-k”), SMA^+^ mesoderm cells (Fig. 3l-l”) and AFP^+^ endoderm cells (Fig. 3m-m”), as efficiently as wild-type H9 did.

#### TET1 Deficiency does not impair directed neural differentiation induced by SMAD inhibitors

**Figure 4:**
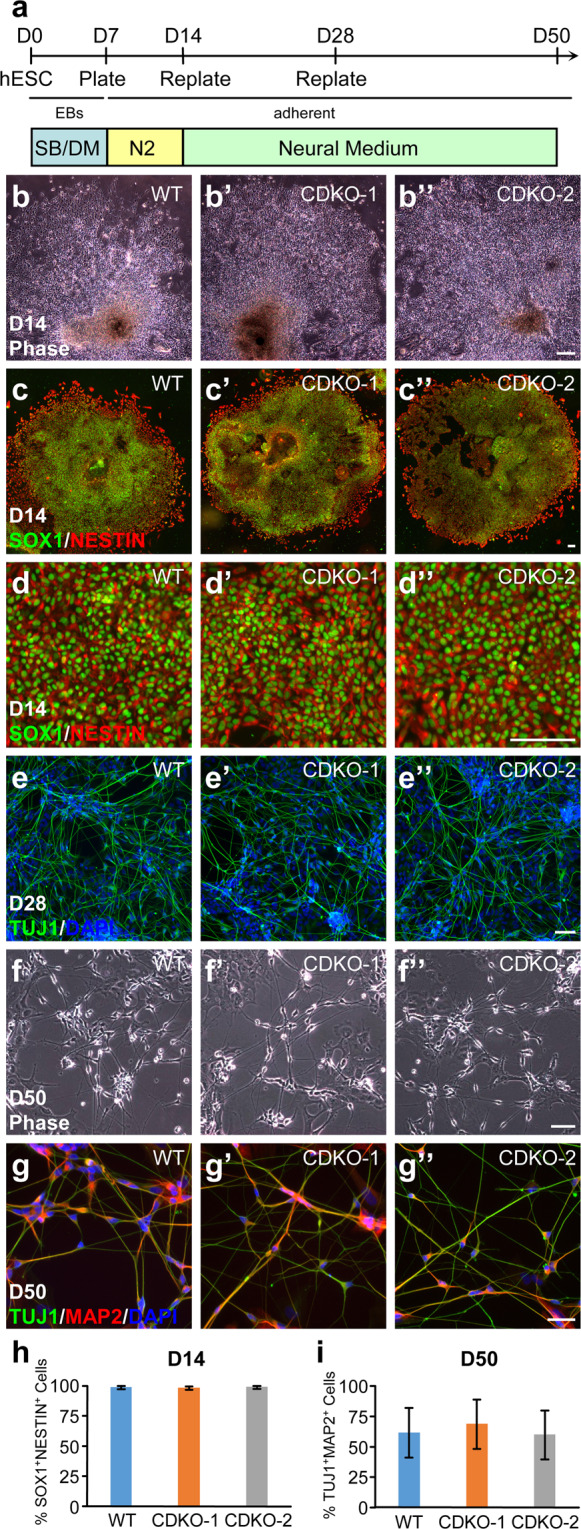
Normal neural differentiation of TET1-deficient hESCs under dual SMAD inhibition. (**a**) Diagram of neural differentiation protocol using the two SMAD inhibitors SB431542 (SB) and dorsomorphin (DM). EB, Embryoid Body. N2, N2 supplements. (**b**-**d”**) Phase contrast images (**b**-**b”**) and immunostaining for neural lineage markers SOX1 and NESTIN (**c**-**d”**) of EB-derived colonies at D14 from the indicated hESC lines. Representative colonies stained in (**c**-**c”**) were magnified in (**d**-**d”**) accordingly. (**e**-**e”**) Immunostaining of the pan-neural marker TUJ1 at D28. (**f**-**g”**) Phase contrast images (**f**-**f”**) and immunostaining of TUJ1 and MAP2 (**g**-**g”**) of mature neurons at D50. (**h**) Quantification of SOX1^+^NESTIN^+^ neuroectoderm cells at D14 from 3 independent experiments. (**i**) Quantification of TUJ1^+^MAP2^+^ mature neurons at D50 from 3 independent experiments. Bars, 100 µm.

Inhibition of the SMAD pathways by SB431542 (SB) and dorsomorphin (DM) drives the differentiation of hESCs to neural lineage^32–34^. As previous studies in mESCs have shown that Tet1 deficiency impairs neuroectoderm differentiation, we differentiated wild-type H9 and the two TET1-deficient mutant lines to EBs, which were treated with 10 µM SB431542 and 5 µM Dorsomorphin for seven days and cultured in DMEM/F12 with N2 supplements for another seven days (Fig. 4a). At Day 14 of differentiation, morphologically indistinguishable colonies of neuroectoderm cells were observed (Fig. 4b-b”). These colonies uniformly expressed the neuroectoderm markers SOX1 and NESTIN to very similar degrees (Fig. 4c-d”). Quantification revealed no significant difference in the percentage of SOX1 and NESTIN double positive cells among the three lines of cells (Fig. 4h). Further differentiation of the replated neuroectoderm cells in Neural Medium (Fig. 4a) generated TUJ1^+^ cells at Day 28 in WT and TET1 mutant lines (Fig. 4e-e”). At Day 50 of differentiation, all three lines of cultures contained neurons of normal morphology (Fig. 4f-f”) and expressed both the mature neuronal marker MAP2 and the pan neural marker TUJ1 (Fig. 4g-g”). There was no significant difference in the percentage of MAP2 and TUJ1 double positive cells among the three lines of cultures (Fig. 4i).

#### TET1 Deficiency impairs the neural differentiation of hESCs in morphogen-free condition

**Figure 5:**
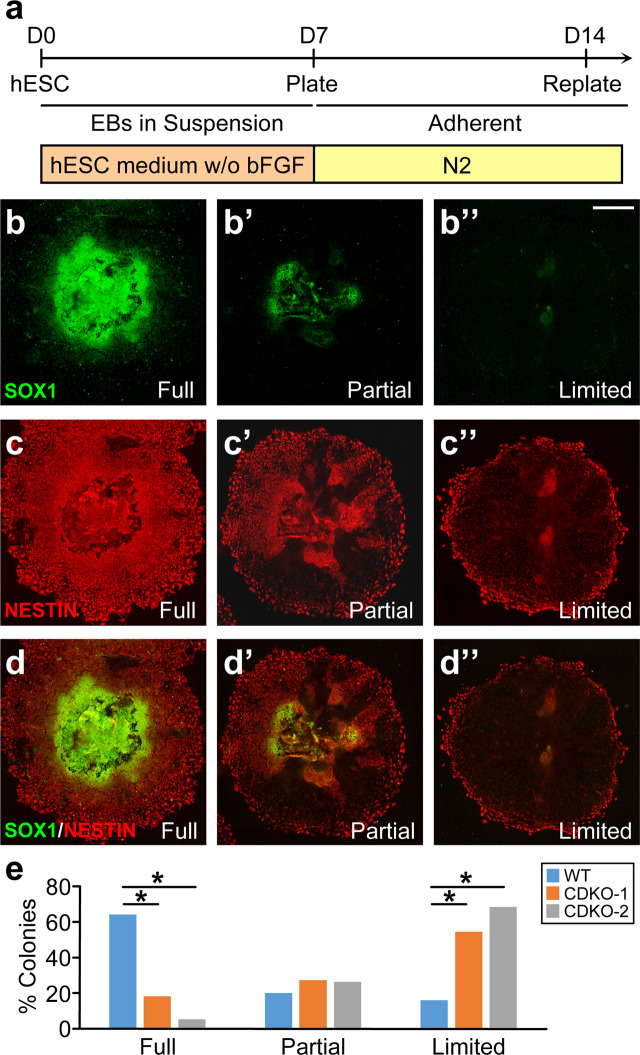
Impairment at the early stage of neural differentiation of TET1-deficient hESCs under the morphogen-free condition. (**a**) Protocol for the differentiation of embryoid bodies (EB) to early neural lineage under morphogen-free condition from day 0 to day 14. N2, N2 supplements. (**b–d”**) Three types of neuroepithelial colonies were classified by the percentage of SOX1^+^ NESTIN^+^ cells among all cells within individual colony, representing the completeness of neural lineage commitment. Full, colonies with a SOX1^+^NESTIN^+^ percentage >70% (**b–d** for separate channels and merged image); Partial, colonies with a SOX1^+^NESTIN^+^ percentage between 20–70% (**b’**-**d’**); Limited, colonies with a SOX1^+^NESTIN^+^ percentage <20% (**b”**-**d”**). Bar, 500 µm. (**e**) Percentage of each type of colonies formed at D14 by WT and the two TET1-deficient lines of H9 hESCs. *p < 0.001; Fisher’s exact test, n = 40 colonies for each cell line.

As dual SMAD inhibition directs hESCs to neuroectoderm^32,33^, we examined the differentiation of TET1-deficient hESCs in a morphogen-free condition to understand whether the intrinsic differentiation program of hESCs is affected by TET1 mutations. H9 or TET1 mutant hESCs were dissociated to single cells, which were aggregated in Aggrewells in hESC media without bFGF and cultured in suspension for 7 days and plated for adherent culture in N2 media for another 7 days (Fig. 5a). At Day 14, each colony formed by an EB contained varying percentages of SOX1 and NESTIN double positive neuroectodermal cells, reflecting the completeness of neuroectoderm commitment in different colonies. We assigned these colonies to three groups according to the percentage of SOX1 and NESTIN double positive cells among all cells in the colony with the following criteria (Fig. 5b-d”). A colony with a percentage more than 70% was assigned to “Full” (Fig. 5b-d), that between 20% and 70% was assigned to “Partial” (Fig. 5b’-c’) and that with less than 20% was assigned to “Limited” (Fig. 5b”-d”). TET1 deficiency significantly reduced the percentage of “Full” colonies and increased the percentage of “Limited” colonies, without significantly changing the percentage of “Partial” colonies (Fig. 5e).

**Figure 6:**
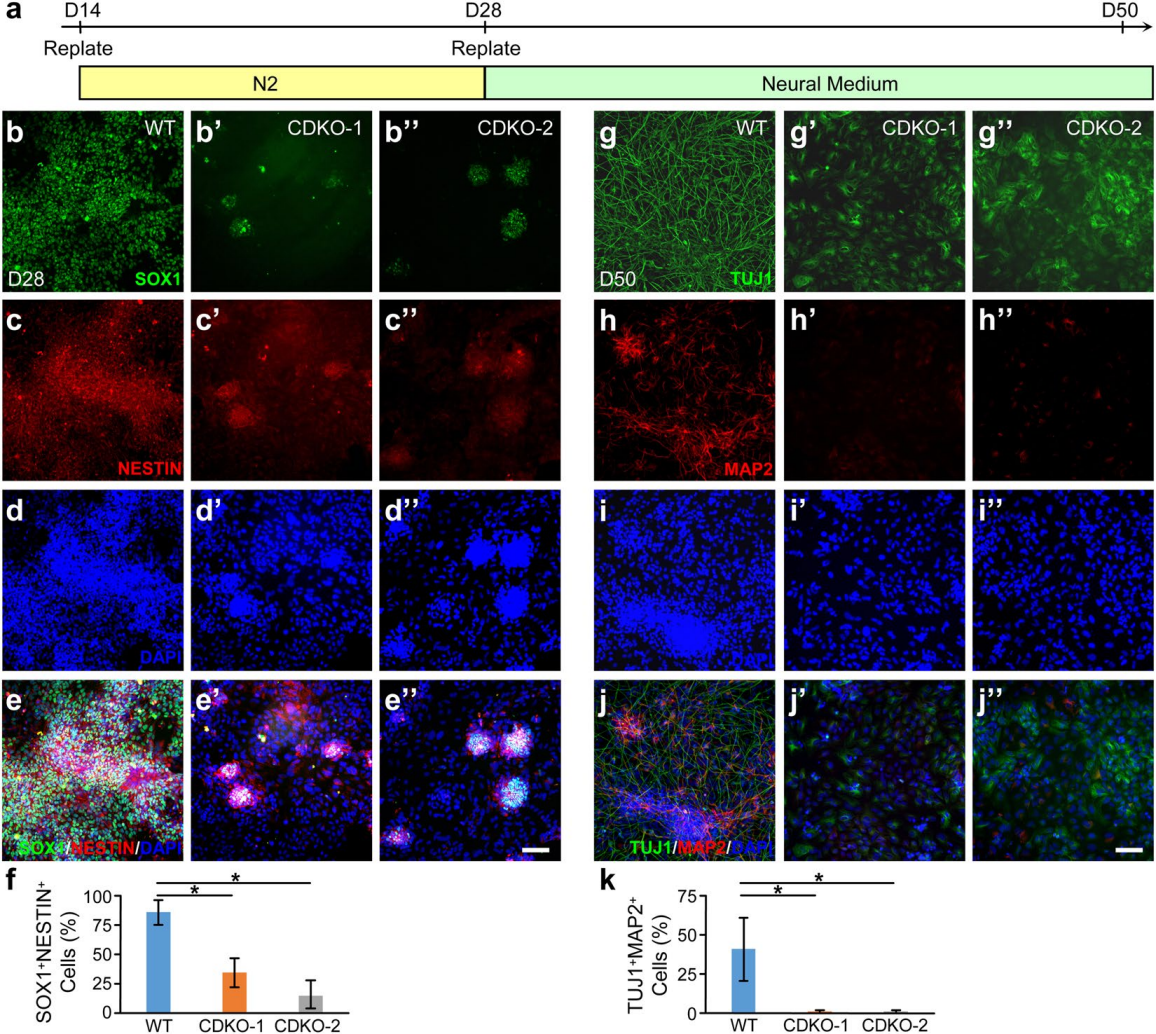
TET1-deficient hESCs cannot be differentiated to neurons in the morphogen-free condition. (**a**) Protocol for the differentiation of neuroepithelial cells to neurons in morphogen-free condition. N2, N2 supplements. (**b–f**) At day 28 of differentiation, neuroectoderm cells generated from wild-type (**b–e**) or TET1-deficient (**b’-e”**) H9 hESCs were costained for SOX1 (**b–b”**), NESTIN (**c–c”**), and DAPI (**d–d”**). Merged images (**e–e”**) were used to quantify the percentage of SOX1 and NESTIN double positive cells among all DAPI^+^ cells (f). *p < 0.001, Student’s *t*-test, n = 3. (**g–k**) At day 50, cells differentiated from wild-type or TET1-deficient H9 hESCs were co-stained for the pan-neural marker TUJ1 (**g–g”**), mature neuronal marker MAP2 (**h–h”**), and DAPI (**i–i”**). Merged images (**j–j”**) were used to quantify the percentage of MAP2 and TUJ1 double positive neuron among all DAPI^+^ cells (**k**). *p < 0.001. Student’s *t*-test, n = 3. Bars, 100 µm.

The colonies were replated and further differentiated in N2 medium until day 28 and then in neural medium until day 50 (Fig. 6a). At Day 28, SOX1 and NESTIN co-staining of the wild-type H9 and TET1 mutant hESCs (Fig. 6b-e”) showed a dramatic decrease of SOX1^+^NESTIN^+^ neuroectodermal cells in TET1 mutant lines, in comparison to the wild-type (Fig. 6f). At day 50, cells were costained with the pan-neural marker TUJ1 and the mature neuronal marker MAP2, together with DAPI (Fig. 6g-j”). Cultures differentiated from wild-type H9 contained numerous neurons with elaborate processes (Fig. 6g), many of which were positive for MAP2 (Fig. 6h). In contrast, the two TET1 mutant lines generated TUJ1^+^ cells with no neuronal processes (Fig. 6g’-g”) and no significant MAP2 expression (Fig. 6h’-h”). Quantification of MAP2 and TUJ1 double positive cells showed a significant reduction from 41 ± 20% in the wild-type to 1 ± 1% and 1 ± 1% in the two TET1 mutant lines, respectively (Fig. 6k).

#### TET1 Deficiency reduces PAX6 expression and hydroxymethylation of PAX6 promoter

**Figure 7:**
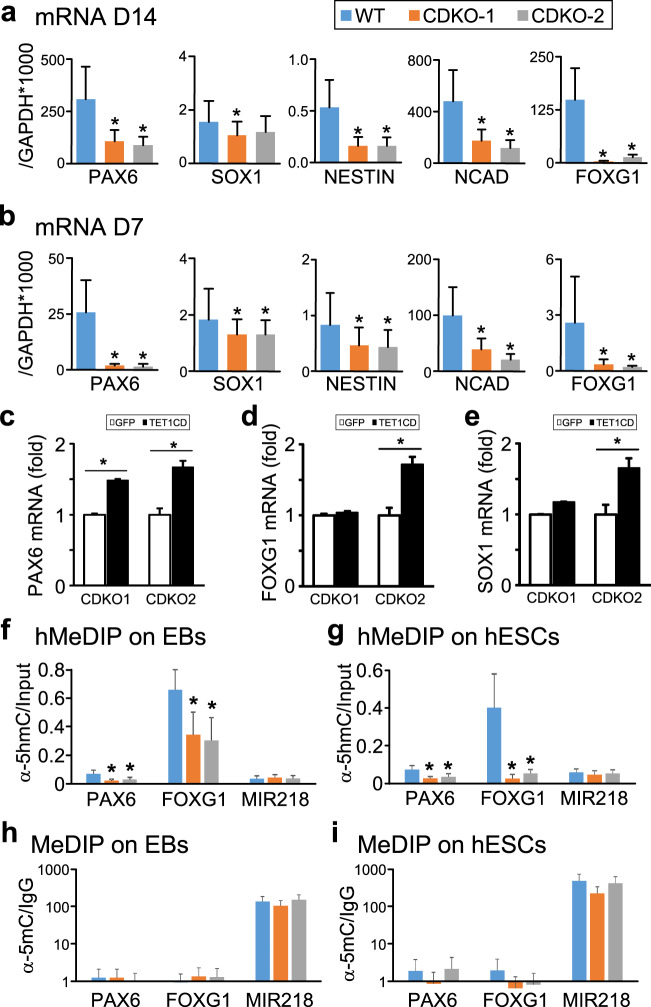
Impaired neural differentiation of TET1-deficient hESCs is accompanied by decreased 5hmC content in PAX6 promoter. (**a**,**b**) qRT-PCR measurement of the expression levels of the indicated neural lineage markers in cells differentiated from wild-type (WT) or TET1-deficient (CDKO-1 and CDKO-2) H9 hESCs under the morphogen-free condition at day 14 (**a**) and day 7 (**b**). *p < 0.05, paired Student’s *t*-test, n = 3. (**c–e**) The two TET1 mutant hESC lines (CDKO1 and CDKO2) infected with lentiviruses expressing GFP or FLAG-TET1CD were differentiated in the morphogen-free condition. Embryoid bodies at day 10 of differentiation were analyzed by qRT-PCR for the expression levels the neuroecdoderm genes PAX6 (**c**), FOXG1 (**d**) and SOX1 (**e**). *p < 0.05, paired Student’s *t*-test, n = 6. (**f**,**g**) hMeDIP measurement of 5hmC content at the promoter region of PAX6, FOXG1 or miR218, a gene unrelated to early neural differentiation, in EBs formed under morphogen-free condition (**f**) or in undifferentiated hESCs (**g**). *p < 0.05, Student’s *t*-test, n = 3. (**h**,**i**) MeDIP measurement of 5mC content at the promoter of PAX6, FOXG1 or miR218 in EBs formed under morphogen-free condition (**h**) or in undifferentiated hESCs (**i**).

To understand why TET1 mutations impaired the intrinsic ability of hESCs to differentiate to neural lineage, we measured the expression levels of several key early markers of neural differentiation, such as PAX6, SOX1, NESTIN, N-Cadherin and FOXG1, during differentiation in morphogen-free condition. At D14, expression levels of most of these marker genes were significantly reduced in both TET1 mutants, except that a significant reduction of SOX1 expression was only observed in one TET1 mutant (Fig. 7a). It confirms a systematic defect in neural lineage commitment. To understand the origin of these defects, we measured the expression of these genes in EBs at D7 and found significant reduction in the expression of all these marker genes (Fig. 7b). The most dramatic decrease was the expression of PAX6, which was expressed about 20 folds less in TET1 mutants than in WT EBs.

To examine whether reduction of these genes is indeed caused by the loss of TET1 catalytic activity, we overexpressed FLAG-tagged TET1 Catalytic Domain (TET1CD) or GFP with lentiviruses in the two lines of TET1-deficient hESCs (CDKO1 and CDKO2) (see Fig. 2i). These hESCs were differentiated in the morphogen-free condition. Expression levels of neuroectoderm genes, such as PAX6, FOXG1 and SOX1, in embryoid bodies at day 10 of differentiation were significantly rescued in CDKO2, and partially in CDKO1 (Fig. 7c–e). The partial rescue in CDKO1 may be caused by many factors, such as variable expression of the viral transgene due to random genomic integration and stochastic effects exerted by the morphogen-free condition on the differentiation of cells.

As PAX6 acts as a master transcription factor to initiate neuroectoderm fate commitment in hESCs differentiation and early human development^30^, we examined whether the hydroxymethylation of PAX6 promoter was affected by TET1 mutations. Using hMeDIP assay, fragmented genomic DNA isolated from EBs at day 7 was immunoprecipitated with anti-5hmC. The amounts of PAX6 promoter in the immunoprecipitates were measured by qPCR. There were significant decreases in the levels of 5hmC in PAX6 promoter in EBs from TET1 mutants, as compared to the wild-type (Fig. 7f). Consistent with the decreased expression of FOXG1 at day 14 (Fig. 7a) and day 7 (Fig. 7b), 5hmC levels in the promoter of FOXG1 were also significantly reduced in TET1 mutants at the EB stage (Fig. 7f). As a control, no significant change in hydroxymethylation was found in the promoter of miR218, a gene unrelated to early neural differentiation (Fig. 7f). Surprisingly, decreased hydroxymethylation of the PAX6 promoter and FOXG1 promoter was observed in undifferentiated hESCs harboring TET1 mutations, in comparison to wild-type H9 (Fig. 7g). MeDIP assay showed that levels of 5mC on PAX6 promoter and FOXG1 promoter were very low and not significantly different among EBs (Fig. 7h) or undifferentiated hESCs (Fig. 7i) with or without TET1 mutations. Expression levels of PAX6 and FOXG1 were too low to be reliably detected by qRT-PCR in wild-type H9 and TET1 mutant hESCs.

#### Reduced expression of PAX6 and SOX1 in Teratomas formed by TET1-deficient hESCs

**Figure 8:**
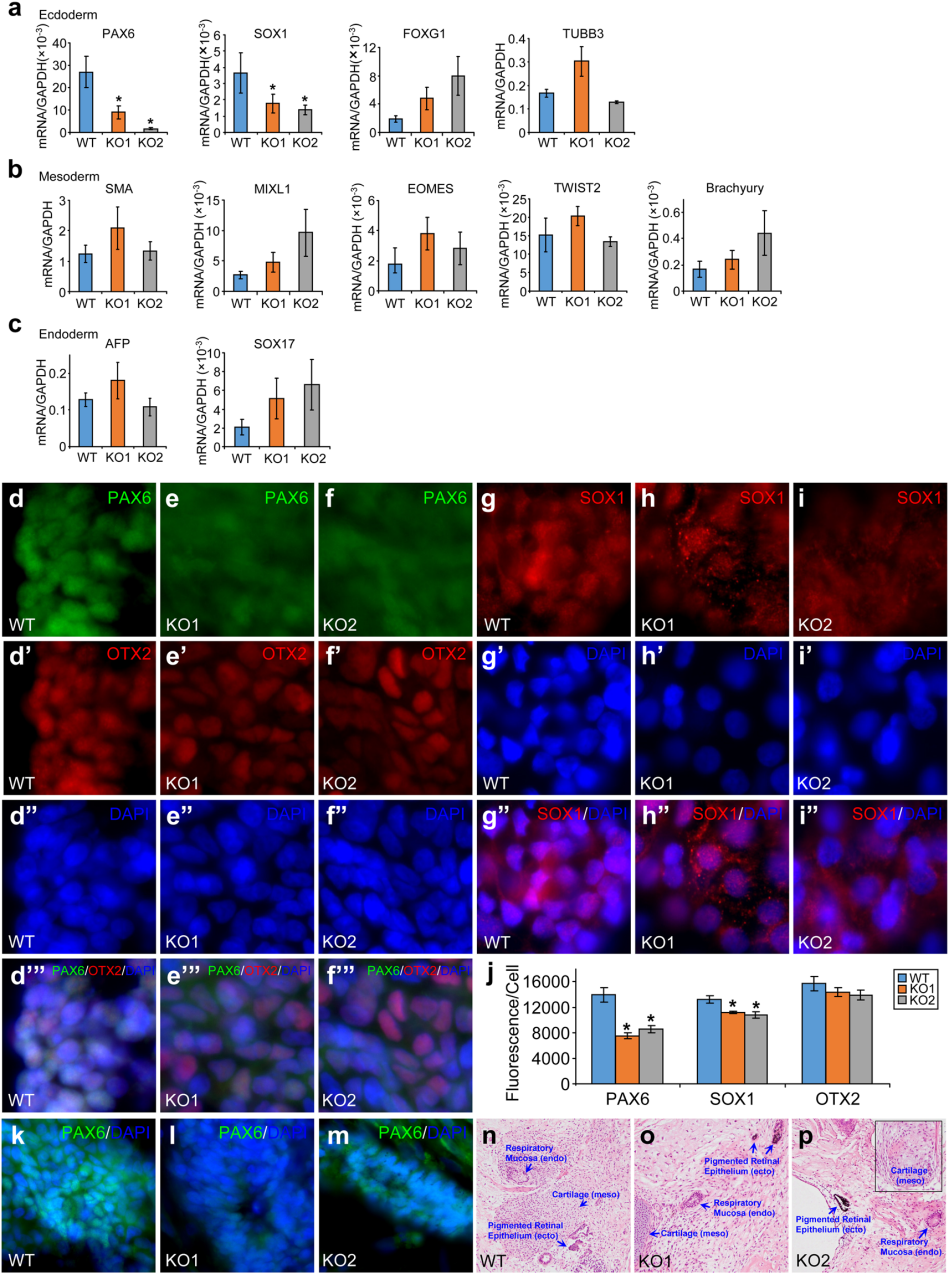
Reduced expression of PAX6 and SOX1 in teratomas formed by TET1-deficient hESCs. (**a**–**c**) Quantitative RT-PCR measurement of the expression levels of marker genes for ectoderm (**a**), mesoderm (**b**), or endoderm (**c**) in total RNA isolated from teratomas formed by TET1-deficient (KO1 and KO2) or wild-type (WT) H9 hESCs. *p < 0.05, vs. WT, n = 8–14. No significant difference between KO1 and KO2. (**d**–**j**) Cryostat sections from these teratomas were costained for PAX6, OTX2 and DNA (**d–f’”**) or for SOX1 and DNA (**g–i”**). Average fluorescence intensities per cell for PAX6, SOX1 and OTX2 in these sections were quantified (**j**). *p < 0.05, vs. WT, n = 12. No significant difference between KO1 and KO2. (**k–m**) PAX6^+^ neural tube-like structures in cryostat sections from these teratomas. (**n–p**) H&E staining of paraffin sections of these teratomas. ecto, ectoderm; meso, mesoderm; endo, endoderm.

To substantiate these *in vitro* findings, we performed teratoma formation assays on the two TET1-deficient lines and their parental wild-type H9 hESCs. In total RNA isolated from these teratomas, we found that the expression levels of PAX6 and SOX1 were significantly decreased in the teratomas formed by the two TET1-deficient hESCs (Fig. 8a). Expression levels of other ecdoderm genes such as FOXG1 and TUBB3 (Fig. 8a), mesoderm genes (Fig. 8b) and endoderm genes (Fig. 8c) were not significantly changed by the loss of TET1. To confirm the findings on PAX6 and SOX1, we performed immunostaining on cryostat sections from the teratomas. The levels of PAX6 (Fig. 8d–f, j) and SOX1 (Fig. 8g–j) fluorescence intensities were indeed significantly reduced in teratomas formed by the two TET1-deficient hESCs. As a control, fluorescence intensities of OTX2 were not significantly different in these teratoma sections (Fig. 8d’-f’, j). Despite the reduction in PAX6 expression, PAX6^+^ neural tube-like structures were found in cryostat sections of teratomas generated by the two TET1-deficient hESCs and the parental wild-type H9 hESCs (Fig. 8k–m). H&E staining of paraffin sections of these teratomas showed the presence of tissues of all three germ layers, including pigmented retinal epithelium, which was derived from neuroectoderm (Fig. 8n–p). It confirms the formation of neural tube-like structures in TET1-deficient teratomas.

## Discussion

In this study, we generated TET1-deficient H9 hESCs by introducing a stop codon in its catalytic domain, which reduced 5hmC level to 30% of that in the wild-type H9 cells (Fig. 2). TET1 deficiency did not significantly change pluripotency in terms of marker gene expression and the ability of the hESCs to differentiate to cells of all three germ layers in the standard serum-containing condition (Fig. 3) and teratoma formation assays (Fig. 8). Thus, our study is consistent with the earlier study showing that loss of Tet1 in mice does not significantly compromise pluripotency and development^25^.

Instead, TET1 deficiency impaired the intrinsic ability of hESCs to differentiate to neuroectoderm (Fig. 5) and neurons (Fig. 6) in a morphogen-free condition. The impairment was accompanied by the dramatically decreased expression of PAX6 and FOXG1 in embryoid bodies at day 7 of differentiation, which persisted in neuroectoderm cells at day 14 (Fig. 7). Reduced expression of PAX6 and SOX1, though not FOXG1, was confirmed in TET1-deficient teratomas (Fig. 8). The consistent decrease in PAX6 expression in multiple independent assays is more meaningful, as PAX6 is a key regulator in the differentiation of hESCs to neuroectoderm and in the development of neuroectoderm in early human embryos^30^. Overexpression of PAX6 in hESCs drives the exit of pluripotency to neuroectoderm, while PAX6 knockdown blocks the differentiation of hESCs^30^. In TET1-deficient hESC, there was a significant decrease in 5hmC level at the promoter of PAX6 and FOXG1 (Fig. 7g), even though PAX6 and FOXG1 expression in hESCs is too low to be reliably detected. Thus, in TET1-deficient hESCs, reduction of 5hmC on PAX6 promoter poises the gene for reduced expression in embryoid bodies and neuroectoderm when bFGF was withdrawn to allow the differentiation of hESCs.

In the presence of the two SMAD inhibitors (SB431542 and dorsomorphin), differentiation of TET1-deficient hESCs was unaffected (Fig. 4). One possibility is that the reduction in PAX6 expression in TET1-deficient hESCs might not be severe enough to block the strong neural differentiation signals provided by dual SMAD inhibition^33^. Direct ablation of PAX6 expression by RNAi blocks the differentiation of hESC to neuroectoderm even in the presence of double SMAD inhibitors^30^. Thus the hypohydroxymethylation of PAX6 promoter in TET1-deficient hESCs may contribute to the decreased expression of PAX6, but not to the level that can block neural differentiation driven by dual SMAD inhibition. By generating TET1-deficient H9 hESCs, this study showed that TET1 played a key role in the hydroxymethylation of PAX6 promoter and the expression of PAX6 *in vitro* (Fig. 7) and *in vivo* (Fig. 8), as PAX6 critically regulates the differentiation of hESCs to neuroectoderm. Loss of TET1 catalytic activity did not significantly compromise pluripotency in hESCs, but greatly impaired the intrinsic ability of hESCs to differentiate to neuroectoderm. Indeed, overexpression of TET1 catalytic domain rescued the defects in 5hmC levels (Fig. 2i–k) and neural differentiation (Fig. 7c–e) in TET1-deficient hESCs, further demonstrating that the ability of TET1 to catalyze the conversion of 5mC to 5hmC is important to support the differentiation of hESCs to neuroectoderm. The function of human TET1 *in vivo* appeared to be more nuanced, as TET1 deficiency did not prevent the formation of neural tube-like structures and neuroectoderm derivatives, such as pigmented retinal epithelium, in teratomas, despite the significant reduction in PAX6 expression (Fig. 8). Other confounding factors, such as the presence of other TET genes and the stochastic nature of teratoma formation assays, may contribute to the observation.

## Methods

### Construction of the CRISPR plasmid

The TET1-CDKO CRISPR site (GACTTCTGTGCTCATCCCCAC) was designed using the online tool at http://crispr.mit.edu/. The corresponding guild RNA sequence was cloned into pSpCas9(BB)-2A-GFP (PX458, Addgene) following the previously published protocol^35^. Efficacy of this CRISPR site and plasmid were confirmed in 293T cells using Surveyor nuclease assay (Integrated DNA technologies, IDT).

### hESC Culture and gene editing

H9 hESCs were cultured on Mouse Embryonic Fibroblasts (MEF) feeder cells as previously described^36^. Briefly, hESCs were propagated on MEF feeders in hESC medium (DMEM/F12, 20% KOSR, 1x NEAA, 1x glutamine, 1x penicillin streptomycin, 4 ng/ml bFGF) for 7 days and dissociated with 1 mg/ml dispase (Stemcell technologies) to small clumps and reseeded at 1:6 on new MEF feeders. To generate mutations in the catalytic domain of TET1, H9 cells were cultured on matrigel-coated vessels (Corning #354277) in mTeSR1 (Stemcell technologies) medium^37^ and passaged with Accutase (Stemcell technologies) as single cells every 4–5 days. TET1-CDKO CRISPR plasmid (10 μg) was delivered to 1 × 10^6^ H9 hESCs in suspension using Nucleofector 2B (Lonza) with program A23. After 2 days of culture on matrigel, cells were dissociated to single cells and FACS-sorted for GFP^+^ cells, which were seeded on matrigel and cultured for another 10 days. Single H9 colonies were manually picked, dissociated and cultured as individual clones. Genomic DNA was extracted from these individual clones using protease K. A 400 bp region flanking the CRISPR targeting site of TET1 was amplified by PCR (primers listed in Table S1) and sequenced to identify clones with mutations in at least one allele. The PCR fragments were cloned into TA vector and sequenced to determine the exact genotype of mutated clones.

### Spontaneous differentiation in Serum-containing medium

After being cultured on MEF feeder cells for 7 days, hESCs were dissociated to medium size clumps using 1 mg/ml dispase and transferred to T25 non-treated culture flasks (Nunc) to form embryoid bodies (EB) in EB medium (DMEM/F12, 20% KOSR, 1x NEAA, 1x Glutamine, 1x Penicillin and Streptomycin) for 4 days. Medium was changed every other day. ROCK inhibitor (Y27632, 10 µM) was added during the first 2 days post passaging. EBs were then seeded on 0.1% gelatine-coated plates and cultured in serum-containing medium (DMEM/F12, 10% FBS) for another 3 weeks before immunostaining for germ layer-specific markers.

### Neural differentiation

After being cultured on MEF feeder cells for 7 days, hESCs were dissociated with TryLE to single cells. We added 2000 cells to each well of ultra-low attachment 96-Well Round-Bottom Microplate (Corning) and cultured the cells in EB medium. ROCK inhibitor (10 µM) was added during the first 4 days. Two SMADs inhibitors, SB431542 (10 µM) and Dorsomorphin (5 µM), were added in the canonical neural differentiation protocol^34^, but were omitted in the morphogen-free condition. On day 7, EBs were seeded on matrigel-coated plates and cultured in N2 medium (DMEM/F12, 1x N2 supplement, 1x NEAA, 1x Penicillin/Streptomycin). On day 14, cells were dissociated with TryLE to single cells and replated on matrigel-coated plates and cultured in N2 medium. For further neuronal differentiation, dissociated cells were replated on polyornithine/matrigel-coated plates at 2 × 10^4^/cm^2^ in Neural medium (Neurobasal, 1× B27 supplement, 1× Glutamine, 1× NEAA, 1× Penicillin/Streptomycin, 20 ng/ml NGF, 20 ng/ml BDNF, 20 ng/ml GDNF), with the addition of 0.5 mM dcAMP and 2.5 µM DAPT two days later.

### Dot blotting

Measurement of 5mC or 5hmC levels using dot blotting was performed following a previous protocol^28^. Briefly, genomic DNA of hESCs was extracted using QIAamp DNA mini Kit (Qiagen), denatured at 100 °C for 10 min in 0.1 M NaOH, and neutralized with an equal volume of cold 2 M ammonium acetate (pH 7.2). Three microliters of each sample diluted to desired concentration were dotted on nitrocellulose blotting membrane (Amersham, GE healthcare) and subjected to UV-crosslinking after air drying. Membrane was briefly rinsed in TBST (20 mM Tris, 150 mM NaCl, 0.1% Tween 20, pH 7.5), and incubated with blocking solution (3% BSA in TBST) for 1 hr at room temperature (RT), then incubated with primary antibody diluted in blocking solution overnight at 4 °C. On the second day, membrane was washed in TBST for 4 times, 15 min each, and incubated with HRP-conjugated secondary antibody in blocking solution for 1 h at RT, then washed in TBST for 4 times again. ECL substrate (Pierce) was then applied and results were recorded and analyzed in ChemiDoc imaging system (Bio-rad).

### Quantitative RT-PCR

RNA of each sample was extracted using Purelink RNA mini kit (Thermo Fisher) and reverse transcribed using iScript cDNA synthesis kit (Bio-rad). Real-time quantitative PCR was performed using iQ SYBR Green Supermix (Bio-rad) in CFX96 Touch™ Real-Time PCR Detection System (Bio-rad). Primer sequences were listed in Table S1.

### MeDIP/hMeDIP Measurement of 5mC or 5hmC Levels

Measurement of 5mC or 5hmC levels on PAX6 promoter was carried out using MeDIP/hMeDIP^38^. Briefly, Genomic DNA of hESCs was extracted using QIAamp DNA mini Kit (Qiagen), diluted to 300 ng/μl in 100 mM Tris-HCl (pH 8.0), and fragmented to 200–600 bp with Sonic 300 Dismembrator (Fisher) on ice. Immunoprecipitation was performed using MeDIP/hMeDIP kit (Active motif). In each reaction, 0.5 μg fragmented genomic DNA was incubated with 4 μg antibodies against 5mC or 5hmC, respectively, for overnight at 4 °C in the buffer provided in the kits, then precipitated with Protein G magnetic beads following the manual of the kit. Immunoprecipitates were used as templates for PCR amplification of specific DNA sequences covering PAX6 promoter or the control gene miR218. 0.05 μg fragmented genomic DNA was used as input as suggested by the kit. Sequences for the primers used to amplify the genomic DNA fragments in the immunoprecipitates were listed in Table S1.

### Lentivirus-mediated Rescue

FUW-TetO-FLAG-human TET1 CD (catalytic domain, 4252–6411 nucleotides) was constructed and packaged in 293FT cells as described previously^28^. CDKO1 and CDKO2 cells were infected overnight with FUW-TetO-FLAG-hTET1CD or FUW-TetO-GFP with M2rTA at MOI of 30 in the presence of 8 ng/ml polybrene. Next day the cells were washed three times with DMEM/F12 and 1 μg/ml DOX was added into the medium. The cells were further cultured in the presence of 1 μg/ml DOX for 3 days. Genomic DNA was extracted for dot blotting with 5mC or 5hmC. Some cells were split to make EB and cultured in the morphogen-free condition containing 1 μg/ml DOX for 10 days. RNA was extracted for qRT-PCR.

### Immunofluorescence

Immunostaining was performed as previously described^36^. Cells were fixed with 4% paraformaldehyde in PBS for 20 min, permeabilized with 0.1% Triton X-100 in PBS for 30 min at RT, blocked in 3% BSA in PBS for 60 min at RT, and then incubated in primary antibody overnight at 4 °C, secondary antibody for 2 hr at RT. Information on the antibodies used in this study is listed in Table S2. Fluorescence images were acquired on Leica AF6000 inverted fluorescence microscope. Quantification of cells was performed manually using 5 independent frames at 10× magnification or 5 individual colonies for each condition.

### Teratoma formation assays

As described previously^36^, one million hESCs (wild-type H9, CDKO1 or CDKO2 TET1 mutant H9) were mixed with collagen at a 1:1 ratio and were aliquoted to ~10 µl pellets, which were grafted under the renal capsule of each kidney in a SCID mouse (C.B-Igh-1bIcrTac-Prkdcscid/Ros). Both kidneys of a mouse were used and three mice were used for each line of hESCs. Large tumors (~1 cm in size) were found for each hESC line 2–3 months after grafting. Teratom tissues were dissected so that a portion was lysed in Trizol for extraction of total RNA, another portion was fixed in 4% paraformaldehyde for embedding and cryostat sectioning, and a third portion was fixed for 24 h in 10% formalin and processed for paraffin embedding and staining with hematoxylin and eosin for histological identification. All animal work for the teratoma formation assay was performed by Mouse Tumor Model Resource at Roswell Park Comprehensive Cancer Center following the approval of the Institutional Animal Care and Use Committee of Roswell Park Comprehensive Cancer Center. All experiments on animals were performed in accordance with relevant guidelines and regulations.

### Statistics

All statistical tests were performed in R 3.0 (The R Project for Statistical Computing). Error bars represent S.D. (Standard deviation). Indicated statistical tests were separately performed on WT vs. CDKO1 and WT vs. CDKO2, since they were independent comparisons. In qRT-PCR experiments, paired student’s *t*-test were used to minimize the interference of batch-to-batch variation.

## Supplementary information

Supplementary Tables 1 and 2.

## Data Availability

All data generated or analyzed during this study are included in this published article (and its Supplementary Information files) or available from the corresponding author on request.
