# Supplementary material for: TET1 Deficiency Impairs Morphogen-free Differentiation of Human Embryonic Stem Cells to Neuroectoderm
Source: Sci Rep. 2020 Jun 25;10:10343. doi: 10.1038/s41598-020-67143-x (PMC7316867; doi:10.1038/s41598-020-67143-x)
Supplement: Supplementary file 1 — Supplementary Tables 1 and 2. [file 41598_2020_67143_MOESM1_ESM.pdf]

Supplementary Information

TET1 Deficiency Impairs Morphogen-free Differentiation of Human Embryonic Stem Cells to Neuroectoderm

Hanqin Li[1], Zhixing Hu[1], Houbo Jiang[1], Jiali Pu[2], Ilana Selli[1], Jingxin Qiu[3], Baorong Zhang[2], Jian Feng[1,*]

[1]Department of Physiology and Biophysics, State University of New York at Buffalo, Buffalo, NY 14203

[2]Department of Neurology, Second Affiliated Hospital, College of Medicine, Zhejiang University, Hangzhou, China.

[3]Department of Pathology and Laboratory Medicine, Roswell Park Comprehensive Cancer Center, Buffalo, NY 14263.

Table S1

Sequences of Primers Used in the Study. Related to Experimental Procedures

Experiment	Gene	Forward	Reverse
Mutation screening	TET1	CACCCTGCCTTCCCTTGTAA	CTGCCTCCCAGCAAAAACAC
qRT-PCR	SOX1	TACAGCCCCATCTCCAACTC	GCTCCGACTTCACCAGAGAG
	PAX6	TAAGGATGTTGAACGGGCAG	TGGTATTCTCTCCCCCTCCT
	Nestin	GCGTTGGAACAGAGGTTGGA	TGGGAGCAAAGATCCAAGAC
	FOXG1	AGGAGGGCGAGAAGAAGAAC	TCACGAAGCACTTGTTGAGG
	N-cadherin	TCCTGATATATGCCCAAGACAA	TGACCCAGTCTCTCTTCTGC
MeDIP/hMeDIP	PAX6	GTACCCTTCGGCTGCTTCAT	AATGCCGCACGATTAGGACA
	MIR218	GTTCCGTTTCCATCGTTCCA	ACCTTGACTCTGACCAGTCG
	FOXG1	CCCCCTTATCCAAAGCTGCG	ACTTTTCCGAGTCGGCCTTG
Teratomas	TUBB3	CGGTGGTGGAACCCTACAAC	AGGTGGTGACTCCGCTCAT
	SMA	CTGAGCGTGGCTATTCCTCCGTT	GCAGTGGCCATCTCATTTTCA
	AFP	GCAGAGGAGATGTGCTGGATTG	CGTGGTCAGTTTGCAGCATTCTG
	TWIST2	TCTGAAACCTGAACAACCTCAG	CTGCTGTCCCTTCTCTCGAC
	Brachyury	ACAGCCAGCAACCTGGGTA	CATGCAGGTGAGTTGTCAGAA
	SOX17	GAGCCAAGGGCGAGTCCCGTA	CCTTCCACGACTTGCCCAGCAT
	EOMES	CTGGCTTCCGTGCCCACGTC	CATGCGCCTGCCCTGTTTCG
	MIXL1	TACCCCGTCTCTTCAACCCT	GCATGCAGAGTCATTGGAGC

Table S2

Antibodies Used in the Study.

Antibody	Catalog #	Vender	Dilution
OCT4	MAB4401	Millipore	1:1000
NANOG	MABD24	Millipore	1:1000
SOX2	MAB4343	Millipore	1:400
SSEA-3	MAB4303	Millipore	1:1000
SSEA-4	MAB4304	Millipore	1:1000
TRA-1-60	MAB4360	Millipore	1:1000
TRA-1-81	MAB4381	Millipore	1:1000
TET1	MA5-16312	Thermo Fisher	1:500
5mC	39649	Active motif	1:1000
5hmC	39769	Active motif	1:1000
AFP	A008	Dako	1:1000
SMA	A5228	Sigma	1:1000
TUJ1	AB78078	Abcam	1:1000
MAP2	sc-74421	Santa Cruz	1:500
PAX6	PAX6	Developmental Studies Hybridoma Bank	1:1000
SOX1	AF3369	R&D systems	1:1000
OTX2	AF1979	R&D systems	1:1000
HRP-conjugated anti mouse IgG	NA931	GE healthcare	1:2000
HRP-conjugated anti rabbit IgG	NA934	GE healthcare	1:2000
Donkey anti-Rabbit IgG (H+L), Alexa Fluor 488	A21206	Thermo Fisher	1:2000
Donkey anti-Mouse IgG (H+L), Alexa Fluor 546	A10036	Thermo Fisher	1:2000
Donkey anti-Goat IgG (H+L), Alexa Fluor 647	A21447	Thermo Fisher	1:2000
